# Physical activity and sedentary behavior perceptions in overweight and obese adults: A systematic review of qualitative study

**DOI:** 10.12688/f1000research.152905.1

**Published:** 2024-07-10

**Authors:** Neha Bora, Vaishali K, Ashwani Verma, Avinash Kumar Bharti, Mukesh Kumar Sinha

**Affiliations:** 1 Department of Physiotherapy, Manipal College of Health Professions, Manipal Academy of Higher Education, Manipal, Karnataka, 576104, India; 2 National Project Officer, United Nation Development Programme, New Delhi, India; 3 Department of Physiotherapy, Indira Gandhi Institute of Medical Sciences, Patna, Bihar, 800014, India

**Keywords:** exercise, sedentary lifestyle, weight gain, beliefs, scoping review

## Abstract

**Background:**

One of the largest hazards to human health is obesity, which is intimately related to sedentarism and physical inactivity. Understanding the perspectives and attitudes of adults with overweight or obesity towards their lifestyle choices related to sedentary behavior and physical activity is essential for mitigating associated health risks.

**Objective:**

This systematic review aims to collate the extent of qualitative research on the perception of sedentary behavior and physical activity among adults with overweight and obesity.

**Methods:**

A comprehensive search of Scopus, PubMed, CINAHL, and Web of Science, databases was conducted, which yielded 2,881 articles. A total of 2591 abstracts were screened, and 45 full-text articles were examined. A total of nine qualitative studies involving adults with overweight or obesity (BMI > 25 kg/m ^2^) were included in this systematic review. Data extraction utilized Rayyan.qcri.org software, and studies were critically appraised using Joanna Briggs’s Institute checklist for qualitative research.

**Results:**

The included studies revealed a diverse array of themes, wherein a few perceived factors reported towards sedentary behavior were lack of awareness about the hazards, mode of relaxation, family commitment, technology use, motivation deficits, and fatigue. Barriers to physical activity encompassed social, cultural, and environmental factors. In contrast, peer support, fitness facility access, accountability, mental health awareness, well-being, and weight management facilitate physical activity involvement.

**Conclusion:**

Perceptions in overweight and obese adults on sedentary living and exercise are intricate and multifaceted. This review provides valuable insights that can inform clinicians and researchers in promoting regular physical activity for adults with overweight and obesity.

## Introduction

Obesity, characterized by chronic adipose tissue inflammation and overeating, leads to sustained fat storage and influences various physiological processes. ^1^ It poses significant risks to human health, playing a major role in the surge of non-communicable diseases. ^2^ As per WHO, one out of every eight individual is obese globally in 2022. ^3^ Notably, between 1980 and 2019, the prevalence of obesity experienced a dramatic increase, surging from 3.2% to 12.2% for men and from 6% to 15.7% for women. ^4^ The Global Burden of Disease in 2015 reported the prevalence of obesity had doubled in more than 70 countries, resulted in 4 million deaths from 1980-2015. ^5^ If these trends persist, it is projected that by 2025, 2.7 billion adults will be overweight, and over one billion will be obese globally. ^4^

In addition to the abundance of endocrine, metabolic, and environmental factors, it is presently understood that the rise of the global obesity epidemic in recent decades is primarily attributed to behavioral factors such as sedentary living and a decrease in overall physical activity (PA), coupled with the consumption of unhealthy diets. ^6^ Individuals who are overweight and obese often exhibit higher levels of sedentary behavior (SB), ^7^ characterized by activities involving minimal energy expenditure (around 1.5 Metabolic Equivalent Units) while sitting or lying down. ^8^ This SB not only contributes to additional weight gain but also leads to reduced PA levels, perpetuating the cycle of obesity. ^8^

Addressing obesity’s impacts can be significantly alleviated through lifestyle modifications, including increased PA, adopting a nutritious diet, ensuring sufficient sleep, and reducing SB. ^2^ The World Health Organization promotes healthy eating and regular exercise as effective and accessible means to prevent and manage overweight and obesity and to mitigate the burden of non-communicable diseases. ^2^ Despite knowing the importance and health benefits of PA, individuals with overweight and obesity are shown to be less physically active than adults with normal weight. ^9^ Thus, to address sufficient PA in this population, it is important to understand the perspective of individuals who are overweight or obese regarding their lifestyle. Systematic reviews are conducted on factors facilitating and limiting PA in various resource settings and low-middle-income countries. However, little is known about the perceptions of PA and SB at the global level from different countries and the differences noted in these countries. This work contributes to filling the gap. Qualitative studies can be considered appropriate tools to capture expressive information about beliefs, values, sentiments, and motives ^10^ in understanding the viewpoints of individuals who are overweight and obese. This review of qualitative study aims to map and compile current literature on perceptions of adults with overweight or obesity towards PA and SB, facilitating a comprehensive understanding of their perspectives.

## Methods

This systematic review was not pre-registered. We chose Arksey and O’ Malley’s ^11^ framework (2005) for its flexibility, enabling the inclusion of a diverse range of study designs and broad concepts within the systematic review process. We followed the procedural guidelines detailed in the methodology manual provided by Levac et al. ^12^ and quality assessment using Joanna Briggs Institute ^13^ checklist. The preferred reporting items for Systematic Reviews and Meta-analysis Guidelines-Scoping Review guidelines guided this review. ^14^ The checklist details are uploaded in Figshare as extended data document 1. ^29^

### Eligibility criteria

This systematic review focused on research pertaining to perception, behavior, or attitude toward PA and SB among adults with overweight/obesity ( Table 1).

**Table 1.** Eligibility criteria.

Characteristics	Inclusion criteria	Exclusion criteria
Study design	Qualitative studies	Quantitative studies, all reviews, reports, commentary, editorials, and conference abstracts
Participants	Individuals with overweight/obesity Age 18-60 years	Studies targeting disease-specific population (e.g., arthritis, diabetes, CVD*, pregnancy, childhood obesity, bariatric surgery) Cognitively unfit Population < 18 years
Outcomes	Physical activity, physical inactivity, sedentary behaviour, sedentary lifestyle, perception, attitude, and beliefs	
Publication	Published in English language	Languages other than English

### Information sources and search

An extensive search was conducted across four electronic databases- Medline via PubMed, Web of Science, Scopus, and CINAHL- employing a varied array of keywords to maximize inclusivity and sensitivity in identifying relevant research published from 2000-2024. The search strategy was developed in consultation with the Information Specialist and subject matter expert (NB, VK, MK, AV, and AKB) through multiple rounds of brainstorming sessions. The final search in all the databases has been conducted by two independent authors (NB and MK). The search term were “overweight,” “obese,” “obesity population,” “adult,” “older adult”, “adult population”, “Physical activity,” “physical inactivity”, “exercise”, “sedentary behaviour”, “sedentary lifestyle”, “perception”, “attitude, and beliefs”, “qualitative studies”, “mixed methods study”, “perception study”. The search strategy was developed with the belief that it would capture all relevant information related to PA and SB perception in adults with overweight/obesity. Apart from electronic searches, manual exploration of the reference lists of all incorporated studies was executed to ensure the inclusion of eligible and pertinent research.

### Selection of sources of evidence

**Figure 1.**
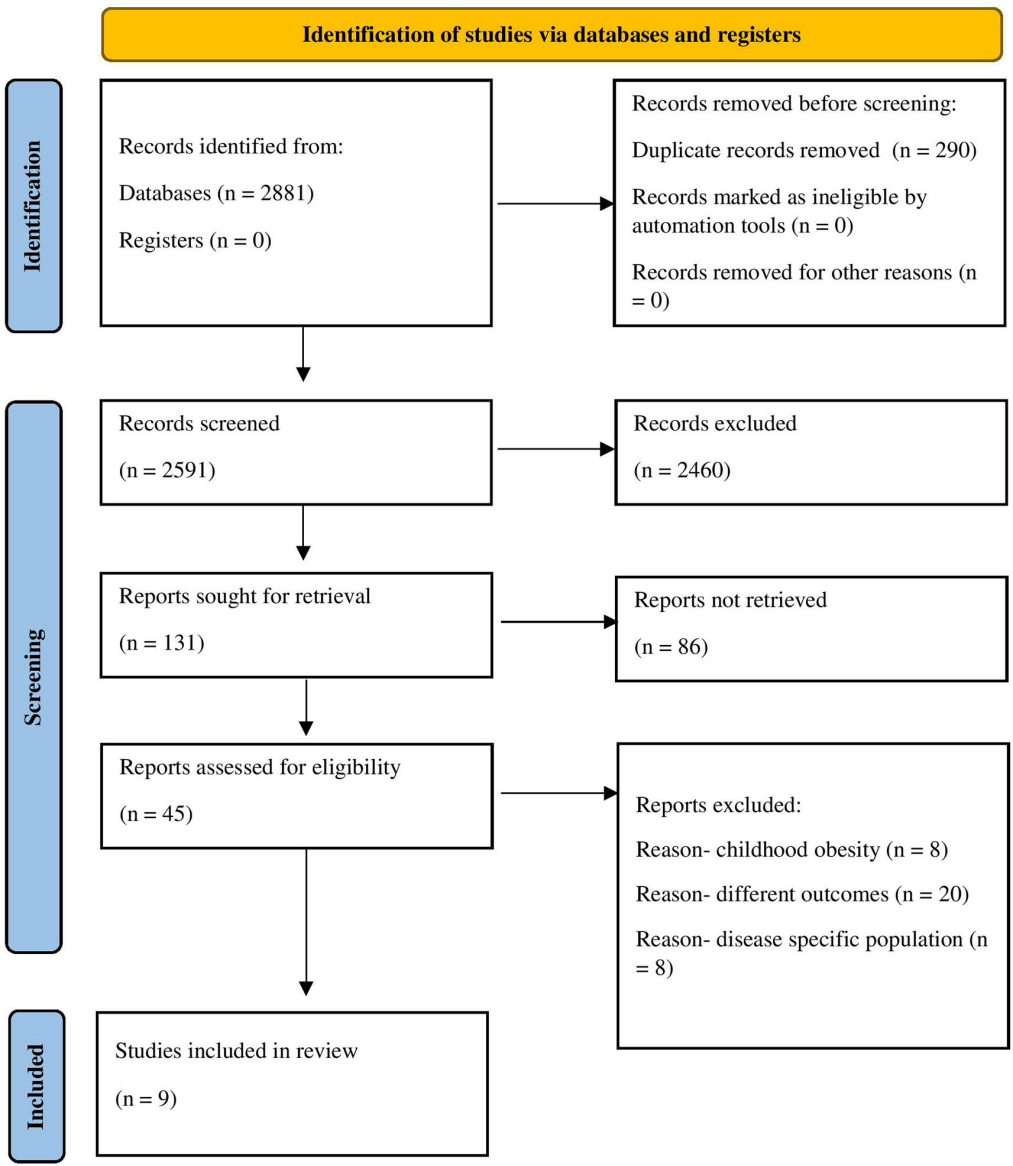
PRISMA flow diagram. *From:* Page MJ, McKenzie JE, Bossuyt PM, Boutron I, Hoffmann TC, Mulrow CD, et al. The PRISMA 2020 statement: an updated guideline for reporting systematic reviews. BMJ 2021;372:n71. doi: 10.1136/bmj.n71.

Two independent reviewers (NB and MK) conducted the eligibility evaluation using Rayyan.qcri.org software. ^15^ A total of 2881 records were identified, wherein 290 records were duplicated and deleted. A systematic title and abstracts (Ti-Ab) screening for 2591 records was conducted by NB and MK based on the predefined criteria. Among the 45 articles selected for full-text screening, nine met the eligibility criteria and were included in the review. Disagreements between the reviewers were resolved through consensus for two studies, with the reviewers (VK, AV, and AKB) stepping in when consensus was difficult to reach. The search and screening procedures, adhering to PRISMA guidelines, are visually depicted in Figure 1.

### Data extraction

The authors (NB, VK, MK, AV, and AKB) prepared a data extraction tool, which underwent pilot testing to ensure relevance. Adjustments were made to the data sheet to meet the review’s objectives. Subsequently, two independent reviewers (NB and MK) manually extracted the data from included studies. Table 2 provides a comprehensive overview of study characteristics for each included study, encompassing details such as authors, publication year, study setting, sample size, and population demographics (age and cultural identification).

**Table 2.** Characteristics of all the included studies.

Authors/year	Study setting/country	Sample size	Population	Age	Cultural identification
Garcia D., et al. (2017) ^17^	Arizona, USA	14 (male=14)	Overweight	18-64 years	Hispanic
Guess N. (2012) ^18^	London, UK	30 (male=5, female=25)	Overweight and obese	18-more than 60 years	Multi-ethnic
Barua, et al. (2022) ^19^	Kolkata, India	18 (male=9, female=9)	Overweight	25-54 years	Multi-ethnic
Alvarado M., et al. (2015) ^20^	Bridgetown, Barbados	17 (female=17)	Overweight and obese	27-34 years	Afro-Caribbean
Baruth M., et al. (2014) ^21^	South Carolina, USA	28 (female=28)	Overweight	25-50 years	African Americans
Joseph R., et al. (2017) ^22^	Arizona, USA	25 (female=25)	Obese	24-49 years	African American
Warren T., et al. (2018) ^23^	South Carolin, USA	32 (female=30)	Overweight and obese	45-65 years	African American
Warbrick I., et al. (2016) ^24^	Auckland, New Zealand	18 (males=18)	Sedentary overweight	28-72 years	Māori
Ashton L., et al. (2015) ^25^	Callaghan, Australia	26 (male=26)	Overweight	18-25 years	Multi-ethnic

### Critical appraisal

Each included study underwent critical appraisal using Joanna Brigg’s Institute critical appraisal tool for Qualitative studies ^16^ by two independent authors (NB and MK). The assessment revealed methodological quality scores surpassing seven (7/10), indicating a good classification. Any discrepancies identified during the quality assessment process were addressed through extensive discussions and mutual agreement among the reviewers. In cases where disagreements persisted, the authors (VK, AV) were consulted to facilitate resolution. The quality assessment of the articles included is summarized in Table 3.

**Table 3.** JBI quality appraisal score for included nine studies.

Author/year	Philosophical perspective and methodology	Question and methodology	Methodology and methods to collect data	Method and data analysis	Method and interpretation	Researcher culture orientation	Influence of researcher	Representation of participants	Ethical approval	Relation of conclusion to analysis
**Garcia et al., 2017**	1	1	1	1	1			1	1	1
**Guess N**	1	1	1	1	1			1	1	1
**Barua et al., 2022**	1	1	1	1	1			1	1	1
**Alvarado et al., 2015**	1	1	1	1	1	1	1	1		1
**Baruth et al., 2014**	1	1	1	1	1	1		1	1	1
**Joseph et al., 2018**	1	1	1	1	1			1		1
**Warren at al., 2018**	1	1	1	1	1			1	1	1
**Warbrick et al., 2016**	1	1	1	1	1	1		1	1	1
**Ashton et al., 2015**	1	1	1	1	1			1	1	1

## Results

A total of nine studies from different countries were included in this scoping review. Perception of PA in adults with overweight/obesity from all nine studies is represented in Table 4, where (+) and (-) indicate positive and negative understanding, respectively.

**Table 4.** Perceptions towards physical activity.

Author/year	Key themes-PA perception
Guess N. (2012) ^18^	Health- related benefits (+) Lack of knowledge (-) Weight loss as primary motivation towards PA (+) Feeling different or self-conscious (-)
Alvarado M, et al. (2015) ^20^	Barriers to PA-gender specific norms, socioeconomic factors, lack of self-efficacy. (-) Facilitators to PA-social support and pressure, access to opportunities, and positive experiences. (+)
Baruth M, et al. (2014) ^21^	Personal barriers (lack of motivation, not having fun, comments on body size, injuries, and health conditions) (-) Social barriers (responsibilities and lack of partner) (-) Environmental barriers (safety, expensive gym, and lack of access to facilities) (-)
Ashton L, et al. (2015) ^25^	Factors motivating men to engage in PA- Physical appearance, social inclusion, physical and mental health and sport or performance (+) Barriers to PA- Busy lifestyles, social and emotional factors, and cost and limited access as logistic factor (-)
Warbrick I, et al. (2016) ^24^	Motivators for PA-interacting with peers, sense of accountability, constructive competition, and overall well-being. (+) Barriers for PA-adulthood priorities, technology, expensive and crowdy gym culture, and lack of knowledge. (-)
Joseph R. et al. (2017) ^22^	Lack of awareness-not aware about intensity, duration, and frequency, unable to differentiate between PA and exercise. (-) Factors enhancing self-efficacy-previous experiences with PA (+,-), barriers to PA (-), and self-identified strategies to overcome them. Strategies to promote PA-goal-setting, tracking, and rewarding.
Garcia D, et al. (2017) ^17^	Barriers to PA-limited access to safe places, long work hours, and fatigue. (-) Knowledge of PA-staying healthy for family and reducing risk for chronic illnesses. (+)
Barua S, et al. (2022) ^19^	Lack of PA as a risk for various illnesses. (+) Factors influencing engagement in PA-good physical and mental health (+), lack of time (-), automated transportation (-), and poor self-efficacy (-)

Adults with overweight/obesity demonstrated a substantial awareness of the benefits of PA and the risks associated with a sedentary lifestyle. Despite this awareness, individuals tended to neglect regular PA, influenced by various factors. Notable contributors to this behavior included a lack of motivation, lack of self-efficacy, time constraints, excessive use of technology, and responsibilities related to work, family, and community. On the positive side, studies also highlighted factors encouraging PA, such as social support, weight loss goals, positive past experiences, overall health benefits, and access to facilities.

Further detailed findings are framed under the following headings.

### PA perception with health benefits

Adults consistently link their perceptions of physical activity to both health benefits and physical appearance. They consistently view PA as the primary approach to achieving physical fitness, maintaining mental stability, and reducing the risk of chronic illnesses. Among nine studies, seven studies reported PA needs to be encouraged to ensure living healthy and disease-free17-20,22,24,25. Witnessing friends and family members coping with long-term chronic conditions serves as a significant motivator for them to engage in PA and take charge of their health. ^20^ The relationship between PA and mental health is closely intertwined with mindfulness, happiness, stress reduction, and overall improved state of mind. ^19^^,^^25^ A sense of accountability, the role of being providers for the family and community, and the desire to serve as healthy role models for the children became the few most typical reasons for achieving physical fitness. ^22^

### The role of attitudes in PA engagement

An individual’s attitudes, deeply rooted in personal beliefs, perceptions, and motivators, act as influential factors that can either propel or hinder an individual’s commitment to maintaining an active and healthy lifestyle. Those with a positive attitude or experience view PA as a beneficial and enjoyable pursuit, which significantly influences PA promotion. ^22^ When individuals derive enjoyment and satisfaction and witness positive outcomes such as weight loss from engaging in PA, it contributes to a heightened sense of self-efficacy. ^22^ These positive associations foster a more favorable attitude towards exercise and motivate participants, encouraging sustained participation. Conversely, negative attitudes, such as finding exercise boring, monotony in routine activities, or experiencing dissatisfaction due to lack of perceived benefits, can lead to demotivation and hinder the adoption of regular PA. ^21^

### Factors influencing PA engagement

The present scoping review identified several facilitators and barriers among adults with overweight/obesity concerning PA participation. Commonly reported facilitators included perceived health benefits for oneself and family, social interaction with peers, ^17^^,^^20^^,^^22^^,^^24^^,^^25^ weight loss goals, ^18^^,^^20^^,^^22^^,^^25^ improved mental well-being, and increased self-esteem and confidence. ^22^^,^^25^ Physical appearance and attractiveness were potential motivators for young men. ^25^ Gender norms were observed to limit opportunities for exercise in women. ^20^ Other barriers identified in this population included lack of safety, ^21^ motivation, ^17^^–^^22^^,^^24^^,^^25^ limited resources, time constraints, ^17^^–^^22^^,^^24^^,^^25^ overuse of technology, fatigue, ^17^^,^^21^ laziness, family responsibilities, and shifting cultural and social values. Moreover, increased weight and associated injuries were observed to limit PA among this population. ^21^ Recognizing these factors can help design practical and sustainable approaches to encourage PA in adults with overweight/obesity.

### Effective strategies to promote PA

This review revealed few commonly reported time-saving strategies to promote PA within this population. Practical approaches identified were implementing workplace activities, ^22^ setting specific achievable goals, and incorporating social changes like engaging in activities with friends and family during leisure time. ^17^^,^^20^^,^^22^^,^^24^^,^^25^ Moreover, altering social activities to prioritize active pursuits and reduce sedentary time emerged as an important strategy. Participating in gym activities alongside peers may seem appealing; however, its costliness, need for a trainer, and inconvenient timings render it a subjective option for many. ^24^ Also, self-reported interventional approaches towards PA promotion, such as the use of technology where text messages or phone calls could be used as reminders for an individual to move around and also help them to track and compare the process throughout. ^17^ Rewards and reinforcements or gift cards or tangible products (like clothing) were a few incentives reported by women who were overweight. ^22^ Women also mentioned walking, dancing, active jobs, and joining Zumba classes with friends as a few strategies to engage themselves, along with the guilt of missing those classes. ^20^ Social media and public health campaigns were credited for enhancing the knowledge about obesity-related diseases. ^17^

### Sedentary behavior perception

Among the nine studies, three of them ^17^^,^^21^^,^^22^ investigated perceptions and attitudes regarding sedentary behavior, as well as the various factors influencing sedentarism. A common theme emerging across these studies are represented under the following headings.

### Sedentary behaviour-ignorance and unawareness

Adults with overweight/obesity often exhibit a lack of awareness regarding sedentarism and its associated risks, primarily stemming from a deficiency in knowledge. However, few adults perceived sedentarism as a prolonged sitting period with minimal movement, ^23^ whereas few perceived it as a risk factor for obesity, various chronic diseases, and other health deteriorating conditions. ^19^ Adults who are overweight often experience SB as a form of relaxation after extended working hours and intertwined with increased leisure time and activities.

### Sedentary behavior perception as a habit or daily routine

This review identified the contributors to SB, specifically among adults with overweight/obese, categorizing them into personal, social, and environmental factors. Personal factors include daily routine activities such as prolonged periods of inactivity in bed, excessive television viewing, seeking enjoyment and relaxation, allocating personal time, and experiencing reduced productivity post-work. Social factors revolve around social events and the influence of friends and family contributing to SB. ^21^ Both work and home environments play significant roles in influencing SB, with prolonged sitting for relaxation or meeting job demands being prominent factors. Age emerges as a frequent influencer of SB, often leading to decreased PA levels and prolonged sitting. ^23^

### Cultural and social shift: Impact on sedentary behavior

In recent decades, cultural and social lifestyles have significantly changed, leading to a surge in SB characterized by increased sitting and relaxation. The pervasive influence of technology in society has further amplified this trend, resulting in decreased levels of PA. ^23^^,^^25^ Additionally, social factors play a crucial role, including the prevalence of desk-bound jobs, leisure time sitting, and heavy reliance on vehicles, ^23^^,^^25^ computers, and cell phones at home and in the workplace, ^23^ all contributing substantially to increased sedentary lifestyle in this demographic group.

### Strategies to minimize sedentary behavior

It is paramount to implement strategies aimed at breaking sedentary patterns in adults with overweight/obesity to foster healthier lifestyle habits. These strategies include incorporating regular breaks during work hours, integrating PA into daily routines, transforming social activities into more active behaviors, utilizing feedback and prompts as reminders, ^17^^,^^23^ and seeking social support to sustain these changes. ^23^ Workplace incentives can play a significant role in enhancing PA behavior and reducing SB. Among these strategies, an initial focus should be on raising awareness about the adverse effects of a sedentary lifestyle. Additionally, addressing the impact of technology-driven SB and implementing diverse approaches to adapt to prevailing work cultures are crucial steps. ^23^^,^^25^

## Discussion

This review aimed to gather insights into the perceptions of adults with overweight/obesity regarding PA and SB. By collating diverse perspectives related to PA and SB, our objective was to comprehensively understand the emerging concerns. Key findings emerged across the multiple themes, including factors influencing motivation and limitations to PA, positive and negative attitudes, and various strategies fostering consistent engagement in PA.

Commonly identified facilitators influencing PA in adults with overweight/obesity are health benefits, overall physical fitness, attractive body image, social support, positive past experiences with exercise, weight loss goals, increased self-esteem, and confidence. These factors were similar to a few studies reported in a systematic review conducted in 2021. ^26^ The importance of social support from family members or peers in encouraging and facilitating participation in PA has been highlighted, similar to the findings of a review conducted in 2023. ^27^ The social aspect of PA makes it more feasible and enjoyable and promotes regular engagement. This review also reports barriers that hinder PA, like time constraints, lack of motivation, lack of interest, fear of injuries, increased weight, societal judgment, familial obligations, negative previous experiences, and occupational responsibilities.

A significant proportion of adults with overweight lack familiarity with the term “sedentary,” associating it with prolonged sitting, low PA, relaxation, and leisure pursuits. ^23^ O’Donoghue et al.’s ^28^ 2016 systematic review examined factors related to SB in adults similar to this review study, including reading, driving, work-related sitting, cell phone use, and television watching. SB was linked to perceived fatigue and stress while inversely related to perceived health benefits. This review identified urbanization, technological advancements, and increased reliance on motorized work as key drivers for SB, compounded by prolonged periods of sitting during leisure activities or due to work demand. Awareness of associated health risks and effective intervention strategies remains lacking, necessitating attention from researchers and clinicians.

Most studies on this topic have been carried out in developed countries, emphasizing the impact of modernization and mechanization on lifestyle factors. Conversely, research conducted in developing nations has concentrated on issues such as limited access to exercise equipment and facilities, as well as a lack of social support. Safety concerns, particularly among women in developing countries, have also been highlighted. ^21^ Studies have observed a trend toward increased use of technology among young adults, which correlates with reduced PA and a more sedentary lifestyle. ^24^^,^^25^ Across all studies, a common theme emerges the necessity of social or peer support and adopting time-efficient approaches to promote physical activity.

Several studies have delved into the multifaceted factors influencing obesity, focusing on diet, food habits, and nutrition alongside PA. In a study in 2015, it was reported that some adults who were overweight perceived healthy nutrition as a potential alternative to PA. ^20^ Additionally, research has identified various obstacles to adopting healthy eating habits, such as insufficient knowledge about nutritious foods, challenges in preparing healthy meals, and the pervasive availability of unhealthy food options. ^21^^,^^25^ Moreover, an unsupportive social environment has been highlighted as a significant barrier to maintaining a healthy diet. ^21^^,^^25^ Particularly, women often face dilemmas regarding whether to prioritize purchasing healthier albeit more expensive food or opting for cheaper, less nutritious alternatives. ^21^

This systematic review benefits from its comprehensive screening of a substantial number of abstracts and articles, providing an extensive mapping of perceptions regarding PA and SB among adults with overweight/obesity. This systematic review focuses exclusively on qualitative studies, offering valuable insights into subjective experiences. Additionally, the review assessed the quality of articles, identifying high-quality materials for inclusion, thus ensuring rigor in the analysis. However, this study has a few limitations as the quantitative aspect of the topic was not analysed. Furthermore, researcher bias may influence the interpretation of the findings, which may limit their broader applicability.

A range of attitudes influencing the willingness and unwillingness of adults with overweight/obesity to engage in PA were uncovered, which highlights the importance of addressing psychological barriers and promoting positive self-perception as part of efforts to promote PA. Strategies to promote PA and reduce SB were identified, offering a roadmap for interventions to foster engagement and adherence among this population. Leveraging technology to improve adherence to regular PA (e.g., virtual coaching platforms, goal-setting mechanisms using fitness trackers and apps) can be explored in further research.

## Conclusion

The intricate and diverse perspectives held by adults with overweight/obesity, particularly concerning their PA and SB, offer valuable insights for clinicians and researchers aiming to promote regular PA. Additionally, the study results address the unique challenges faced in workplace environments when adopting sustainable PA. Implementing cost-effective approaches is equally imperative to make interventions accessible and feasible for individuals dealing with overweight/obesity.

## Data Availability

All data underlying the results are available as part of the article, and no additional source data are required. *Reporting guidelines* Figshare: Preferred Reporting Items for Systematic Reviews and Meta-Analyses extension for Scoping Reviews (PRISMA-ScR) Checklist for Physical Activity and Sedentary Behavior Perceptions in Overweight and Obese Adults: A Qualitative Review; Figure. https://doi.org/10.6084/m9.figshare.25997038.v1. ^29^ Data are available under the terms of the Creative Commons Attribution 4.0 International license (CC-BY 4.0).
