# Supplementary material for: Physical activity and sedentary behavior perceptions in overweight and obese adults: A systematic review of qualitative study
Source: F1000Res. 2024 Jul 10;13:787. [Version 1] doi: 10.12688/f1000research.152905.1 (PMC11316175; doi:10.12688/f1000research.152905.1)
Supplement: Peer review history

# Reviewer report: Reviewer response for version 1

**DOI:** 10.5256/f1000research.167716.r302702

**Published:** 2024-08-09

**Authors:** Dr Doss Prakash S

**Affiliations:** 1 Department of Community Physiotherapy, MGM Institute of Physiotherapy, Aurangabad, Maharashtra, India

The systematic review have highlighted multifaceted factors leading to overweight and obesity in adult population.  This review will be an valuable clinical tool for the health care professionals for clinical decision making process. And also provides convincing evidence to promote physical activity among the over weight and obese adults to prevent any medical complications. 

 Review point:

 The overall article is in line with systematic review findings, but it requires some revision… 

1. Authors need to be specific in the introduction section about the literature gap… currently known and the clinical relevance point
2. Include the prevalence of physical activity inactivity as reported in the current WHO report.
3. As the article is about overweight and obese adults, therefore author needs to justify the need for study for both groups (overweight and obese adults) in the introduction section
4. Were there any specific findings reported in the published literature concerning the inclusion criteria of ages 18 to 60 years in the study? Highlight the key findings.
5. Report the characteristics of the included study, specifically focusing on socioeconomic status, occupation, and behavioral information if it is provided in the paper. If this information is not included, discuss its potential impact.
6. Any specific information related to digital, gender, or age group?
7. Is there any information about readiness to change perception?
8. PA perception with health benefits- this section there is one line …. The relationship between PA and mental health is closely intertwined with mindfulness, happiness, stress reduction, and overall improved state of mind- need clarity.
9. Also, in the same section (PA perception with health benefits), what does the adoption of regular PA mean? 

Are the rationale for, and objectives of, the Systematic Review clearly stated?

Yes

Is the statistical analysis and its interpretation appropriate?

Yes

If this is a Living Systematic Review, is the ‘living’ method appropriate and is the search schedule clearly defined and justified? (‘Living Systematic Review’ or a variation of this term should be included in the title.)

Yes

Are sufficient details of the methods and analysis provided to allow replication by others?

Yes

Are the conclusions drawn adequately supported by the results presented in the review?

Yes

Reviewer Expertise:

Community Health, Public Health, Geriatrics, Women's Health

I confirm that I have read this submission and believe that I have an appropriate level of expertise to confirm that it is of an acceptable scientific standard.

# Reviewer report: Reviewer response for version 1

**DOI:** 10.5256/f1000research.167716.r302707

**Published:** 2024-08-07

**Authors:** Saumya Srivastava

**Affiliations:** 1 Department of Physiotherapy, Nitte Institute of Physiotherapy, NITTE (Deemed to be University), Mangalore, Karnataka, India

**Article Summary**

 The article **"Physical activity and sedentary behaviour perceptions in overweight and obese adults: A systematic review of qualitative study" by Bora N, K V, Verma A et al.**, examines the perceptions of physical activity and sedentary behaviour in overweight and obese adults. The authors conducted a systematic review of qualitative studies to understand the barriers and facilitators influencing physical activity and sedentary behaviours in this population.

**Findings:**

 1. Barriers to Physical Activity: Overweight and obese adults reported several barriers, including physical limitations, lack of motivation, and psychological factors such as body image issues and fear of judgement.

 2. Facilitators to Physical Activity: Support from family, friends, and healthcare providers, along with the availability of suitable environments and resources, were identified as key facilitators.

 3. Perceptions of Sedentary Behaviour: Many participants viewed sedentary behaviour as a habitual and difficult-to-change aspect of their lives, often associated with relaxation and leisure activities.

 4. Intervention Preferences: The participants expressed a preference for personalised, enjoyable, and non-judgmental physical activity interventions.

**Strengths and Weaknesses**

**Strengths**

 1. Comprehensive Review: The systematic review includes a wide range of qualitative studies, providing a broad understanding of the perceptions of physical activity and sedentary behaviour in overweight and obese adults.

 2. Depth of Insights: The qualitative nature of the included studies allows for in-depth insights into the personal experiences and perspectives of the participants.

 3. Identification of Key Factors: The study effectively identifies and categorises the barriers and facilitators to physical activity, which can inform the design of targeted interventions.

 4. Practical Implications: The findings have practical implications for healthcare providers in designing and implementing effective physical activity programs for overweight and obese individuals.

**Weaknesses**

 1. Heterogeneity of Studies: The included studies vary in terms of their methodologies, participant demographics, and contexts, which may affect the generalizability of the findings.

 2. Publication Bias: As the review focuses on published qualitative studies, there may be a publication bias, with positive findings more likely to be published than negative or null results.

 3. Lack of Quantitative Data: The reliance on qualitative data limits the ability to quantify the impact of identified barriers and facilitators on physical activity levels.

 4. Potential for Subjectivity: The interpretation of qualitative data can be subjective, and the review's findings may be influenced by the authors' perspectives and biases.

**Relevance and Positive Comments**

 The article is highly relevant for researchers and  healthcare providers interested in promoting physical activity among overweight and obese adults. By providing a comprehensive overview of the barriers and facilitators, the study highlights critical areas for intervention and support.

**Positive Comment:**

 The systematic review by Bora et al. is a valuable contribution to the field of public health and physical activity research. Its in-depth analysis of qualitative studies provides nuanced insights into the lived experiences of overweight and obese adults, offering a robust foundation for designing effective, empathy-driven interventions to promote physical activity and reduce sedentary behaviour in this population.

Are the rationale for, and objectives of, the Systematic Review clearly stated?

Yes

Is the statistical analysis and its interpretation appropriate?

I cannot comment. A qualified statistician is required.

If this is a Living Systematic Review, is the ‘living’ method appropriate and is the search schedule clearly defined and justified? (‘Living Systematic Review’ or a variation of this term should be included in the title.)

Not applicable

Are sufficient details of the methods and analysis provided to allow replication by others?

Yes

Are the conclusions drawn adequately supported by the results presented in the review?

Yes

Reviewer Expertise:

Sacroiliac joint dysfunction, Low back pain, Mckenzie, Mulligan

I confirm that I have read this submission and believe that I have an appropriate level of expertise to confirm that it is of an acceptable scientific standard.
